# Demography as a Field: Where We Came From and Where We Are Headed

**DOI:** 10.1007/s42650-023-00076-8

**Published:** 2023-07-17

**Authors:** Luca Maria Pesando, Audrey Dorélien, Xavier St‑Denis, Alexis Santos

**Affiliations:** 1 Division of Social Science, New York University, Abu Dhabi, United Arab Emirates; 2 Department of Sociology, McGill University, Montreal, Quebec, Canada; 3 Hubert H. Humphrey School of Public Affairs, University of Minnesota, Minneapolis, MN, USA; 4 Institut National de la Recherche Scientifique (INRS), Montréal, Canada; 5 Department of Human Development and Family Studies, Pennsylvania State University, State College, Pennsylvania, PA, USA

**Keywords:** Demography, Innovations, Pedagogy, Data and methods, Public policy

## Abstract

This essay provides a series of reflections on the current state of demography as seen by four early-career researchers who are actively engaged in aspects of the discipline as varied as research, teaching, mentorship, data collection efforts, policy making, and policy advising. Despite some claims that the discipline is weakening, we showcase the great potential of the field and outline promising pathways and novel directions for the future. In so doing, we critically assess recent innovations in data quality and availability, stressing the need to “revolutionize” the way that demographic methods are taught by adopting a viewpoint that more closely reflects the rapidly changing, or “fast,” nature of global social phenomena such as conflict-related displacements, environmental disasters, migration streams, pandemics, and evolving population policies. We conclude by discussing the relevance of careful demographic analyses for policy making, stressing three main points: (i) the need to make demography more visible and understandable to the public eye; (ii) the importance of engaging and co-creating with local communities to “break” the academic bubble; and (iii) the urge to counteract the spread of misinformation—a phenomenon that has become even more visible in the aftermath of the COVID-19 outbreak.

## Résumé

Cet article propose une série de réflexions sur l’état actuel de la démographie selon le point de vue de quatre chercheurs en début de carrière activement engagés dans certaines branches de la discipline telles que la recherche, l’enseignement, le mentorat, la collecte de données, l’élaboration des politiques et le conseil en matière de politiques. Malgré certaines allégations affirmant que la discipline est en perte de vitesse, nous mettons en évidence son immense potentiel et décrivons les orientations prometteuses et originales qui se profilent. À cet effet, nous évaluons de façon critique les récentes innovations en matière de qualité et de disponibilité des données tout en soulignant la nécessité de « révolutionner » l’enseignement des méthodes démographiques en adoptant un point de vue reflétant plus fidèlement l’évolution rapide des phénomènes sociaux mondiaux, notamment les déplacements de populations liés aux conflits, les catastrophes environnementales, les flux migratoires, les pandémies et l’évolution des politiques démographiques. Nous concluons en discutant de la pertinence des analyses démographiques approfondies nécessaires à l’élaboration des politiques en soulignant trois points essentiels : (i) la nécessité d’améliorer la visibilité et l’intelligibilité de la démographie aux yeux du public, (ii) l’importance de s’engager auprès des communautés locales et de collaborer avec elles pour « percer » la bulle académique, et (iii) la nécessité de lutter contre la diffusion de fausses informations, phénomène qui s’est renforcé dans le sillage de l’épidémie de COVID-19.

## 1 Introduction^1^

It is not uncommon for early-career demographers to be told that the discipline is in decline. Statements that reference decline include “demography is disappearing,” “demography is nothing but a branch of applied sociology,” “demography is simply statistics applied to the study of population phenomena,” and, foremost, “what is demography?” The latter is perhaps the most recurrent question posed to demographers over their careers. Taken together, these claims would seem to suggest that the golden days of demography are gone. Thinking strictly about the demographic and epidemiological transitions, this conclusion would make sense, as almost every country in the world completed the first demographic transition, moving from a high-mortality and high-fertility regime to a low-mortality and low-fertility one (with few exceptions in sub-Saharan Africa), making the core demographic pillars of fertility and mortality less central for explaining population change and composition. But, beyond surface, is this really the case? Are we really witnessing a gradual weakening of the field? The aim of this manuscript is to provide a novel perspective on the discipline, highlighting its potential as well as some promising directions for the future that may help the field reposition itself in light of the current world’s global social challenges. The essay is structured as follows: first, we review discussions showcasing how and why some scholars present the discipline as declining; second, we outline our main argument that demography is instead “alive” and thriving, preserving unique elements that distinguish it from other disciplines and social sciences; third, we outline promising pathways and novel future directions in the realms of theory and methods, data and analyses, and teaching; lastly, we conclude by discussing the relevance of careful demographic analyses for policy making and for informing the general public.

## 2 Background: the “Apparent” Weakening of the Field

Every now and then—pretty much every 15 years—prominent demographers take stock of the “progress” of the field by publishing critical essays on the state of the discipline and outlining pathways for the future. Despite some optimism, a constant thread is the “precarious” and “uncertain” placement of the field within the social sciences, as well as its “marginal” perception within governmental institutions, business, and industry. As early as 1979, Nam published the essay “The Progress of Demography as a Scientific Discipline” in the lead journal of the Population Association of America (PAA) *Demography*, claiming that, despite considerable developments, demography falls in the category of fields that have “probationary status” and is “tenuously established in the scholarly world” (Nam, 1979, p. 485), being widely ignored as a field of knowledge of its own. This would be further confirmed by the fact that “in academia, demographers are ordinarily labeled sociologists or economists” (Nam, 1979, p. 485). The same idea is echoed in Preston’s, 1993 *Demography* essay “The Contours of Demography: Estimates and Projections,” that claimed demography is “a small discipline lacking security in academic bureaucracies and always in need of a *raison d’être*” (Preston, 1993, p. 595).

Some of these same tensions are reflected in today’s academic world, with many demographers hosted in departments of sociology, economics, public health, global health, anthropology, geography, etc., and more institutions offering graduate training in population studies through population studies “options” embedded within other mainstream social-science degrees such as sociology and economics. Reality outside of North America is a little different, with demography more likely to retain status as its own discipline, at least from a teaching and training standpoint. Nonetheless, even in those contexts, demography tends to be seen as part and parcel of another bigger field (e.g., economics and, to a smaller extent, sociology in France, public health in the UK and Puerto Rico, statistics in Italy, just to give some examples), leading many demographers to suffer “identity crises” when trying to locate themselves within a specific discipline (Leone, 2010). Overall, the debate is open and the perception of the discipline—including its identity and future—varies from one region, country, school, and individual to another, but it is far from uncommon to encounter scholars that are concerned about the future of the discipline and its institutions (Tabutin, 2007).

Why do scholars find it so hard to situate demography as a separate field with its own perspectives, approaches, and challenges? What are some possible reasons behind general claims that the field of demography is weakening? In his 2007 critical essay, Tabutin outlined several weaknesses of demography, some of which were already discussed in Preston (1993), Roussel (2004), and Poirier and Piché (1999), such as its relative intellectual isolation among the social sciences, its narrow reach as a relatively small discipline, its excessive compartmentalization—both geographical and intellectual—its inability to move beyond measurement and description, its “statistical extremism,” and its lack of public visibility. Using Tabutin’s words: “Demography has not yet fully emerged from its ivory tower, a position which offers freedom to think but also creates a risk of social and political irrelevance” (Tabutin, 2007, p. 24). Similarly, in Roussel’s opinion: “A major obstacle to forming the identity of demographers comes from a sort of retrenchment syndrome” (Roussel, 2004, p. 239).

There are some other features that may have contributed to general perceptions of demography as a weakening field. First is demography’s insufficient progress in explanation and failure to seek a broad understanding of social change (Burch, 1999; De Bruijn, 1999). By nature, demography is a multidisciplinary subject which emphasizes rigorous data analysis using specific methods accompanied by theory that is often associated with sociology, anthropology, economics, and public health, among others (Leone, 2010). As such, demography is not a “theory-heavy” discipline, yet it is informed by a series of theoretical paradigms such as the Malthusian framework, the epidemiological transition (Omran, 1971), the first demographic transition (Lee, 2003), the second demographic transition (Lesthaeghe, 2010; Zaidl & Morgan, 2017), the third demographic transition (Coleman, 2006; Lichter, 2013), and the life course paradigm (Billari, 2001; Elder, 1998). The lack of a substantial theoretical core does contribute to distinguishing it from other social sciences and reinforcing its “tenuous” status to the lay eye. Relatedly, demographers have always been concerned about data quality, careful measurement, sound methodologies, and precision—a feature which is unique to the field yet, if brought to an excess, may hinder theory-generating efforts. In 1984, Livi-Bacci stressed this aspect, highlighting the risk that demography could remain “a technique rather than a science” (Livi-Bacci, 1984). Second, the field is becoming even more interdisciplinary—an aspect that we see as beneficial—yet some scholars may worry that too much interdisciplinarity may weaken what is unique about demography, i.e., its demography “core.” Third, some of the latest global social phenomena such as environmental disasters and the COVID-19 pandemic have cast doubt onto the reliability of predictive and forecasting methodologies, which have proven inadequate to deal with sudden shocks that call for real-time data, methods, and evidence—not to mention the historical inability to predict the baby boom and the baby bust. Lastly, while formal demography is still an active field, it is becoming more and more “niche,” somewhat contributing to making the field of demography more of an empirical undertaking.

While some concerns are well grounded, with this essay we aim to provide our own perspective on the discipline as early-career researchers and identify what we see as its prominent and unique contributions, both current and future. In so doing, we touch upon important innovations in the realms of theory, data, methods, and pedagogical practices, as well as put forward recommendations for how we see the field moving forward and how, in our opinion, it could increase its relevance and visibility further, especially from a policy angle.

## 3 The Uniqueness of Demography

We claim that demography is nowadays more relevant than ever, as its scope, methodologies, and related data sources have broadened to account for a wider variety of global social phenomena, most of which are rapidly changing and require readily available information. Even in terms of scope, demographers’ research interests have become much more heterogeneous since the 1990s, touching on topics such as gender and sexuality, poverty and inequality, development, migration, health, environment, conflicts, natural disasters, climate change, and more. Methodologically, the focus on aggregate analyses has shifted toward individual-level analyses and, eventually, multi-level analyses, also supported by massive improvements in computing power (Anderson, 2022; Tabutin, 2007). There has also been an increase in causal analyses (both experimental and quasi-experimental), enabled by modern data collection techniques, fieldwork, and dissemination practices (Dodoo et al., 2014; Thomas & Frankenberg, 2015), as well as longitudinal study designs that were not as common in the early days of the discipline, especially across low- and middle-income countries (LMICs).

Moving into the details, there is much that remains unique to demography and can be seen as an opportunity rather than a hindrance. First and foremost is the *population-level perspective* that lies at the core of the field defined, as in Duncan (2008), as descriptions (through, for instance, means, distributions, and rates) and relationships found in the population at large. While this has been criticized in the past as providing a “partial” analytical perspective, it is undeniable that demographers are trained as “big-picture” scholars and, as such, they have a comparative advantage vis-à-vis other social scientists in conducting population-level analyses. Obviously, such analytical perspective may well be complemented with meso- and micro-level analyses (as is often the case), which may be better suited to enrich the picture by complementing description with explanatory efforts (Billari, 2015). When thinking about the value of cross-disciplinary collaborations, we see this feature as one of demographers’ greatest strengths, which should be preserved and further strengthened rather than left behind. This same position was already stressed by Ní Bhrolcháin and Dyson (2007) and further echoed in Duncan’s 2008 PAA presidential address: “My fundamental argument is that demographers should be much more aggressive in promoting their population perspective throughout the sciences” (Duncan, 2008, p. 764) and “a population perspective, and the sampling tools that help to produce it, are much too important and powerful to be kept hidden away from our colleagues in the fields of experimental, clinical, and qualitative research” (Duncan, 2008, p. 781). Duncan (2008) further concluded that, despite population regressions typically failing to provide unbiased estimates of causal impacts, a population-based understanding of causal effects should be demographers’ end goal.

Second, with its solid toolkit and newly emerging sources of data, we strongly believe that demography should retain a *global focus*, devoting more space and consideration to LMICs, as well as to resource-deprived areas in high-income countries (HICs), such as rural areas that are witnessing severe depopulation and high poverty rates. On one hand, LMICs—particularly sub-Saharan Africa (SSA) and Southeast Asia (SA)—are the contexts that are undergoing the most massive and noticeable transformations in terms of changing population structure and composition as driven by fertility and mortality forces. One clear example is India, which in April 2023 reached a population of 1,425,775,850, matching and then surpassing the population of mainland China, and thus becoming the world’s most populous country (UN-DESA, 2023). These are also the countries that are witnessing some of the most drastic transformations in family forms and structures (Pesando and GFC Team, 2019), offering unique opportunities for comparative scholarly research, community engagement, and contact with governments and policymakers that may translate into effective policy interventions. On the other hand, while the demographic transition is complete in HICs and family structures exhibit, on average, less variability relative to LMICs (Castro Torres et al., 2022; Pesando, Barban, et al., 2021a), several pressing sociodemographic dynamics require similar attention, such as rural-to-urban migration, rural poverty, and depopulation (among others)—a series of phenomena that are compounded by broader issues such as low fertility, declining life expectancy, and population aging. The latter are particularly pressing, especially in the US and Canadian contexts (Clark et al., 2022; Simard, 2021; Slack & Jensen, 2020). As such, by “global” focus, we refer to a combination of *international respite* yet with a careful eye devoted to local peculiarities, irrespective of countries’ income category.

Third, while the field is moving more and more toward adopting experimental and quasi-experimental techniques typical of other disciplines such as economics, epidemiology, or political science (Dodoo et al., 2014), we want to stress the idea that “good” demography can (and often does) incorporate a causal framework, yet it can also be largely independent of it. Careful *descriptive analyses* lie at the core of the discipline and demographers are uniquely positioned to conduct them with the highest level of rigor and precision. In fact, while causal analysis is essential to the advancement of knowledge in many areas, “how many” and “how different” are core questions for policymakers, civil society, and academics alike, and there is undeniable value in producing and updating this type of more descriptive knowledge in a rigorous way. This is rather obvious when estimating morbidity and mortality trends (Goldstein & Lee, 2020; McGrail, 2022; Wrigley-Field, 2022), but it also extends to other areas. One such example is a recent work by Boen et al. (2022) estimating the cumulative risks of arrest, probation, and incarceration from childhood through early adulthood, assessing disparities by race/ethnicity, gender, and parental education. Other examples may come from the family domain. For instance, simply stressing the importance of accounting for increases in life expectancy when building measures of age at first marriage in LMICs, or age-standardizing age-sensitive family indicators when conducting comparative research across widely different societies is essential to ensure that comparisons reflect the extent to which changing age structures account for differences within/across countries versus more fundamental social processes (Pesando and GFC Team, 2019). Similarly, when studying trends in educational assortative mating in a context such as SSA, there is intrinsic value in determining whether mating patterns are driven by “mechanical” changes that result from proportionally faster increases in women’s education versus “behavioral” responses related to spousal preferences for educational resemblance (Pesando, 2021). These may seem like trivial considerations, but they are essential for correctly measuring and estimating global social phenomena, and ones that are commonly neglected by other social scientists. Demographers are unique in this kind of thinking.

Fourth, we believe demographers should retain their focus on *cross-national* and/or *cross-unit analyses*, be they communities, neighborhoods, cities, regions, countries, etc. In doing so, we will be able to unveil and reveal the inequalities that exist within and across these units around the globe. Recent scholarship on global family change reveals that cross-national comparative studies are well suited to help scholars identify common structural factors underpinning differences in future generations’ outcomes, including gender, health, and educational dynamics, as well as potential country and regional “exceptionalisms” where these structural factors may follow unexpected patterns that do not necessarily conform to extant theoretical predictions (Batyra et al., 2023). Lastly, while it is mostly true that demographers tend to borrow theoretically from other disciplines, this does not invalidate the existence of a *unique demographic lens* for exploring socio-demographic phenomena which combines—but is not limited to—passion for data and innovation with careful demographic measurement, neat descriptions, attention to detail, and, increasingly, sustained efforts toward complementing descriptive efforts with better explanations.

Overall, we believe that the global challenges of the past two decades—such as the global financial crisis, wars, droughts, famines, environmental disasters, increasing income inequality, epidemics, and pandemics—will make the relevance and uniqueness of demography over the coming years more and more salient, overcoming some of the concerns mentioned in the previous section. Nonetheless, this will require a concerted effort between scholars, teachers, institutions, governments, communities, policymakers, and practitioners. We elaborate on all these points in what follows.

## 4 Current and Future Directions and Innovations

### 4.1 Theoretical Paradigm and Methods

Basic demographic phenomena such as changes in mortality and fertility used to be slowly changing forces, evolving over the course of centuries, if not millennia. Not by chance, the typical demographic paradigm depicts demography as a “slow” discipline, assessing phenomena that change over extended time spans and follow relatively “predictable” patterns, in line with the epidemiological and demographic transitions. This view is still partially correct, at least at the global level. However, if there is something that we have learned over the past two decades it is that demographic change can be more unpredictable and rapidly evolving than commonly conceptualized. As such, in line with Billari (2022), we claim that a shift in paradigm is needed to keep demography a vibrant, exciting, and promising field over the coming years. This shift in paradigm that views demography as a “fast” discipline requires revising demographic theories and methods to keep up with the pace of societal changes.^2^

Demography has accelerated in multiple ways. Consider migration and spatial mobility. International and internal migrations occur every day, from every single corner of the world, being voluntary at times and forced at other (e.g., forced displacements in the wake of the recent Ukraine crisis), and responding very quickly to political events or policy decisions. Similarly, think about major health threats such as the Ebola epidemic, centered primarily in Western Africa, or the Zika epidemic, first reported in Brazil in 2016. These are sudden stressors which have the potential to affect mortality, fertility, family composition, and migration over the course of a year, if not months, drastically shaping the age distribution of the population in ways that traditional demographic methods are not well equipped to manage (Marteleto et al., 2020; Suchar et al., 2018). Similarly, the COVID-19 pandemic has affected the demography of every single population in the world, with each deserving attention (Dowd et al., 2020; Goldstein & Lee, 2020; Verdery et al., 2020). These fast-occurring demographic changes mean that governments’ measurements of denominators are often outdated. This can pose problems such as when trying to estimate excess mortality or case fatality rates during the COVID-19 pandemic (Trinitapoli, 2021; Wrigley-Field, 2022). Not least, the diffusion of digital technologies (so-called digital revolution)—accompanied by the massive increase in computing power—has completely altered the way that socio-demographic phenomena unfold, from fertility to reproductive health, marriage patterns, domestic violence, migration, etc. (Billari et al., 2019; Billari et al., 2020; Köksal et al., 2022; Nobles et al., 2022; Pesando, 2022; Pesando, Rotondi, et al., 2021b; Rotondi et al., 2020).

Demographers and population scientists should fully embrace this paradigm shift of demography from a “slow” to a “fast” discipline and adapt their methods and pedagogical practices accordingly. While the perception that global social phenomena have accelerated is widespread by now—and has even pervaded policy circles and shaped policy discourse successfully, featuring prominently in the EU agenda^3^—the methodological core of the discipline has not evolved at the same pace nor has the way that demographic methods are being taught around the world. Revising the teaching core to reflect this “faster” nature of demography will prove essential to keep the field active and thriving. This is likely to include a sustained intensification of demographers’ involvement in primary data collection in a variety of ways. This transformation is well under way and constitutes a marked shift from the use of government surveys and/or official statistics. However, it requires rethinking how we approach technical and methodological training, as further discussed below.

### 4.2 New Data and Analyses

The workhorses of demographic research data collection have been censuses, household surveys, and administrative datasets. The majority of these have been cross-sectional, especially in LMICs (think about the Demographic and Health Surveys or the Multiple Indicator Cluster Surveys), but over the last four decades there have been progressively more investments in various types of longitudinal datasets. A few key developments in data availability are likely to have strong impacts on the future of the discipline, including a shift toward new research topics and perspectives. In this section, we highlight how longitudinal data have shaped life course research on migration and social mobility, and how big data have allowed researchers to increasingly “nowcast” global social phenomena including crises, epidemics, and population displacements, thus embracing the “fast” paradigm described above. To spur innovation, we also need to further promote efforts to facilitate linkages between demographic data sources and external sources of data that can provide additional contextual information that was not collected at the time of survey. This is especially the case for demographers interested in climate change and other population environment research. Finally, in the age of big data, demographers need to embrace new data sources, which are often not representative, contain repeated measures for individuals, and are more complicated because individuals may be observed for different lengths of time and at different points in time.

#### 4.2.1 Longitudinal and Administrative Data

Life-course research lies at the core of our understanding of how inequalities develop and accumulate over a lifetime (Diprete & Eirich, 2006; Mayer, 2009). This perspective dominates social demographers’ scholarship, thanks to the increased level of complexity and comprehensiveness of longitudinal data. Several longitudinal surveys have been running for decades, such as the Panel Study of Income Dynamics (PSID) starting in 1968 in the USA, the German Socioeconomic Panel (GSOEP) starting in 1984 in Germany, and the British Household Panel Survey (BHPS) conducted between 1991 and 2009 in the UK and with a subsample integrated to the UK Household Longitudinal Survey (Understanding Society) since then. Some other run for decades but then are stopped, such as the Canadian National Longitudinal Survey of Children and Youth (NLSCY), as attrition had become too high to generate meaningful results. This collection of full life-course histories for at least two generations opens the door for multi-generational frameworks of analysis. Similarly, long-lasting genealogical longitudinal surveys allow documenting the similarity in overall life-course dynamics between parents and their children, rather than intergenerational transmission of outcomes measured at one point in time in each generation. Prominent examples include the linked lives, linked trajectories framework of Cheng and Song (2019) relying on the PSID, or the study of within-family reproduction of women’s life course trajectories by Vidal et al. (2020) using the GSOEP. This kind of research is still underdeveloped in LMICs, as the development of extensive longitudinal data is lagging behind. Some noteworthy exceptions include the Malawi Longitudinal Study of Families and Health (MLSFH); the Young Lives International Study of Childhood Poverty in Ethiopia, India, Peru, and Vietnam; the Cape Area Panel Survey (CAPS); the Study on Global Ageing and Adult Health (SAGE); and the Indonesian Family Life Survey (IFLS).

The door is wide open for demographers to study how life course dynamics in the parent’s generation influence life course events in the child’s generation, (i) going well beyond a focus on traditional markers of stratification and socioeconomic status such as education; (ii) building frameworks that consider seriously their interplay with core demographic variables such as fertility, mortality, migration, and couple formation, as in Mare (2011) and Song (2021); and (iii) extending this kind of research to LMICs, as more longitudinal datasets covering multiple generations become available. This type of research does require detailed parental and child life course data, in other words relatively long-lasting, multigenerational panels. Adopting such approaches will come with increased costs and need for additional resources to facilitate data collection and follow-up (and minimize attrition), something that should be conveyed to funding bodies to assure the success of such endeavors.

In a few countries, first and foremost the Nordic countries, and more recently Canada (Denton et al., 2011; Denton et al., 2021; Yoshida et al., 2022) and the USA (for example, Chetty et al., 2014), these types of data are becoming available not through longitudinal surveys, but rather through the increased availability of administrative micro-data such as tax records or other register data. Yet challenges lay ahead, which demographers will need to address in order to leverage the full potential of administrative data. First, access is often restricted due to the sensitive nature of some data, the large processing time necessary to construct and analyze such datasets, and the need to combine data from several different sources. Second, academics are dependent on data collection or processing decisions that are not primarily made based on researchers’ needs. Hence, key variables are often missing from many of those datasets, such as subjective questions, reliable measures of several transitions below the annual level, and accurate geographic identifiers. In addition, administrative decisions can shift the reliability of these records. One example comes from the US Internal Revenue Service (IRS) Migration data. Researchers found that shifts in data production from the US Census Bureau to the IRS resulted in concerning and systemic problems with IRS migration data. This is just one of the many instances that continue to challenge users’ reliance on administrative records despite their increased availability (DeWaard et al., 2022).

Because of these challenges, administrative data may not be a panacea for demographers. High-quality longitudinal data have proven essential for demographers, and many countries have shown sustained ambitions to maintain longitudinal surveys; for instance, a broad range of National Education Panel Study (NEPS) cohort data have been collected since around 2010 in Germany, and in 2009, data collection started for the successor of the BHPS, Understanding Society. Most recently, the US Congress has granted the necessary budget to start collecting data for a new NLSY cohort (2026).^4^ Recently, Young Lives announced the onset of Wave 7 data collection, tracking children’s lives from age 0 to 29. In contrast, in other countries such as Canada, we have observed discontinuation of data collection for some longitudinal surveys,^5^ including the NLSCY and, more recently, the Longitudinal and International Survey of Adults (LISA), and a move toward developing longitudinal data by record linkage and reliance on administrative databases. The Canadian experience will help determine in what ways administrative data combined with cross-sectional surveys will contribute to fill the gap left by the discontinuation of some longitudinal surveys, and how other countries will balance cost-effectiveness considerations driving the use of administrative data with the richness of long-lasting but expensive longitudinal surveys.

#### 4.2.2 Spatial Data

The spatial components of demographic data are often overlooked. This is unfortunate because the addition of geographic data, such as county or other detailed administrative identifiers in demographic datasets, can increase the types of research questions studied, as well as expand the types of study populations and granularity of analysis. For instance, to study the impacts of climate change and other environmental exposures, demographers need to be able to merge traditional sources of data with environmental data sources such as remotely sensed climate data or shape files (geographic outlines) of flood extents. Ideally, each observation in the dataset has corresponding geographic coordinates (latitude and longitude, often with a little error to protect privacy). If such information is not available, identifiers for the most detailed administrative boundaries should be included (Dorélien & Grace, 2022). Alternatively, with the inclusion of geographic identifiers, especially geographic coordinates, populations no longer need to be aggregated based on administrative boundaries. Analyses can be based on environmental features such as populations residing along a river or residing within a certain distance from a pollution emitter.

#### 4.2.3 Big Data

Big data are large datasets that may be analyzed computationally to reveal patterns, trends, and associations, especially relating to human behaviour and interactions, including “digital traces” left by users on the web. Demographers have been at the forefront of the use of big data to study sociodemographic dynamics and the changing demographic landscape in close-to-real time. Despite concerns related to the representativeness of these data, big data have become a crucial tool to capture the “fast paradigm” embedded in rapidly changing—and often unpredictable—demographic dynamics such as migration patterns, population displacements, and health threats, often allowing for greater temporal and spatial granularity relative to more traditional data sources. With all due caveats, we see the massive expansion of big data as a fruitful avenue for future demographic research, especially when complemented with—or assessed against—other sources of data such as sample surveys and alternative methodological approaches, such as text or sentiment analysis. Demographers have highlighted the power of big data and data obtained from digital technologies in a range of domains such as migration (Alexander et al., 2022; Miranda-González et al., 2020; Rampazzo et al., 2021; Vieira et al., 2022), fertility (Billari et al., 2016; Wilde et al., 2020), gender inequality (Fatehkia et al., 2018; Kashyap et al., 2020), intimate partner violence (Köksal et al., 2022), and collection of vital/official statistics (Fudolig et al., 2020; Jahani et al., 2017).

#### 4.2.4 Triangulating Evidence

In line with the aforementioned need to complement different methodologies and data sources, powerful demographic research should blend more effectively quantitative and qualitative approaches (Obermeyer, 1997). This is crucial not only to collect more refined contextual information—for instance, on sensitive topics such as violence, sexual health, abortion, etc.—but, foremost, also to evaluate the many biases inherent in quantitative data collection efforts, especially in low-income and/or disadvantaged countries and/or contexts (Randall and Koppenhaver 2004). Examples include, but are not limited to, cleavages between surveyors and respondents’ socioeconomic status, respondents’ silence on specific topics, and the role of interviewer characteristics in influencing the subject matter (Randall et al., 2013). Successful uses of qualitative methods and/or mixed-methods approaches in demography include work by Randall et al. (2011) trying to unpack cultural constructions of the concept of households in sample surveys in Tanzania; LeGrand et al.’s (2003) study on fertility behavior and insurance effects in Senegal and Zimbabwe; Frye and Trinitapoli’s (2015) study of sexuality and relationship experiences in Malawi; and Frye’s (2017) work on sexual relationships and school dropout in Malawi. Within this realm, another promising direction includes adopting mixed-methods frameworks that combine computational techniques such as text analysis with qualitative methods, as shown by Chakrabarti and Frye (2017) in their study of the Malawi Journals Project focused on informal conversations about HIV and by Hastings and Pesando (2022) in their study of parenting in the USA.

### 4.3 The Pedagogy of the Discipline

Teaching is an essential component for keeping a field vibrant and alive, and it falls to teachers to keep abreast of the latest developments in the field and incorporate them into their curricula and syllabi. Yet there can be a high degree of inertia combined with a sense of fear when deciding to move away from “traditional” ways of teaching a specific subject. This is very often the case for demography, where we observe a significant lag between the fast pace of demographic phenomena and the traditional methods taught to graduate students around the world (with some variations). We claim here that revising the pedagogy of demographic teaching is a crucial step to keep our discipline thriving and up to date. While concepts such as standardization and decomposition remain crucial, the main goal is to embrace methods that parallel the “fast” paradigm now embedded in demography, moving away from some demographic models such as stationary and/or stable population theory, which are rooted in a “slow and predictable” view of the world and rely on assumptions inapplicable to many real-world contexts.

Our second suggestion is to broaden the applicability of basic demographic techniques to areas of study that are different from health, mortality, and fertility. Demography encompasses many domains of life, yet demographic methods tend to be narrowly taught, limiting students’ interests and broad understanding. We encourage teachers to showcase the potential of demographic methods for studying additional phenomena, for instance, in the fields of family formation (e.g., obtaining “marital expectancies” instead of life expectancies, such as Pesando and GFC Team, 2019), intergenerational transfers (e.g., applying multi-state life tables to estimate the amount of time spent as provider/receiver of financial and non-financial transfers, such as in Payne et al., 2019), educational transitions (e.g., applying multi-state life tables to the study of grade-to-grade transitions in school, such as in Breen & Jonsson, 2000), or career patterns (e.g., with estimation methods allowing to quantify the share of individuals achieving a given level of job stability at different stages of their life course, such as in St-Denis, 2021). There is also increasing need to tie demographic teaching to issues of sustainability (e.g., applying formal demographic methods to the study of population aging, dependency, and consumption, such as in Lee & Mason, 2014).

Third, we believe that demographers should incorporate into their curricula more thorough modules on key topics such as social mobility, migration and spatial distribution, population displacements, climate change and environmental disasters, population aging, rural-to-urban migration and rural poverty, and the role of population policies in shaping the socio-demographic landscape.

Fourth, teachers should consider accompanying the teaching of demographic methods with frequent in-class labs that showcase the potential of software such as R and Python for analyzing demographic phenomena. In so doing, teachers will also be able to broaden students’ understanding of commonly used sources of data—which are currently far broader than censuses and sample surveys, including geo-spatial data, big data, etc. Relatedly, the increased use of big and unstructured data from the web or from large administrative datasets, as well as natural language data, makes crucial the inclusion of basic computer science, data science, and database management knowledge in demographic training. This type of knowledge not only is increasingly useful in academic research but is also sought after by non-academic organizations likely to employ population scientists. Furthermore, demographers can make important contributions to the field of data science with our focus on data quality, careful measurement, attention to numerators and denominators, and increasing concerns about transparency, accountability, and replicability. Finally, by relying on a more interactive teaching pedagogy, teachers would also be able to highlight the key importance of data visualization efforts, an essential skill for any successful demographer. Broadly thinking about the importance of effective pedagogy in demography, we believe international training initiatives such as the European Doctoral School of Demography (EDSD), the International Max Planck Research Schools (IMPRS), the Consortium on Analytics for Data-Driven Decision-Making (CAnD3), the Berkeley Workshop in Formal Demography, and the Advanced Spatial Analysis Training Program (Pennsylvania State University and University of California, Santa Barbara)—among others—are even more appreciated and needed.

## 5 The Public Face and Visibility of Demography

In recent years—and even more in the aftermath of COVID-19—demography has been at the forefront of almost every public discussion, from daily-life debates to classical concerns and ongoing speculation about the future of the population. These include discussions about heterogeneity across population groups and the role that migration plays in shaping it, the implications of expanding pro-natalist fertility policies—e.g., the overturning of *Roe vs Wade* in the USA,^6^ and new anti-abortion restrictions introduced in Poland^7^ and Iran (Kokabisaghi, 2017), among others—concerns about population well-being, and increasing mortality among middle-aged adults in high-income societies (Case & Deaton, 2015; Harris et al., 2021; Masters et al., 2018). On top of this, concerns about the politicization of censuses, including in the USA^8^ (Prewitt, 2010) and Canada^9^ (Thompson, 2020), accuracy of demographic analyses, and how the latter are portrayed in the media continue to shape the opinions of those who are the subjects as well as potential beneficiaries of our own research.

Over the last decade, demographers have started to engage with community members to elevate the issues that affect communities themselves. For example, Crowell is a social demographer whose scholarship focuses on residential segregation. Her recent work deals with rent and evictions with a special focus on Fresno, California (Crowell, 2022). In her work, she engages with local organizations such as Faith in the Valley to disseminate research from court records to understand the complexities underlying eviction dynamics in Fresno. By unlocking the data from these records, Crowell and local organizations showed that between 13 and 17% of the evictions are non-rent related, and that nearly in 1 to 5 cases tenants are served notices of termination without an explanation. Thanks to this research, scholars and practitioners can start dismantling the prevalent notion that evictions are due to rent delinquency.

Another example of community-engaged research relates to the estimation of post-disaster mortality. For instance, the issue of deaths attributable to Hurricane Maria continues to be a topic of conversation in Puerto Rico. While government officers stated that 16 deaths had occurred due to hurricane, the sheer magnitude of this disaster led some people to question this official figure. The Center for Investigative Journalism and journalist Frances Robles elevated the voices of communities calling into question this figure. Demographers at Pennsylvania State University communicated with family members, journalists, nurses, and public health professionals to assess the reality in the island and determined that additional analyses were needed. A pre-print published in November 2017 applied basic demographic techniques to death records and found that 1000 deaths had occurred in excess of the patterns observed during the 2010s (Santos-Lozada & Howard, 2017); these estimates were later validated through other methods and published in academic journals (Santos-Lozada & Howard, 2018). The continuous engagement with journalists and members of the Puerto Rican diaspora led to additional questions on whether/how Hurricane Maria had impacted migration dynamics. Again, basic demographic techniques, paired with passenger counts, served as the basis for an analysis that demonstrated that 88,000 passengers had left Puerto Rico in excess of the patterns observed during the 2010s (Santos-Lozada, 2018). This staggering figure indicated that the mobility induced by post-disaster conditions in the last 3 months of 2017 surpassed the overall outmigration observed in the previous year. These are just two examples on how engaging with communities can benefit the study of issues that affect populations at a localized level, which may in turn signal broader issues/concerns experienced and faced by the population.

The breadth of the topics studied by demographers, paired with increasing exposure through social media, community-engaged research, and direct contact with individuals and policymakers, has not been without consequence. For instance, some people leverage research that leans in their favor to develop arguments and counterarguments. As mentioned above, one example comes from the experience of demographers trying to ascertain the level of deaths after Hurricane Maria. A government officer seeking to “minimize” the issue of death tolls compared the number of deaths from September 2017 to those of January 2017 and concluded that the number of deaths was similar. While the arithmetic that underlies this statement was correct, the comparison of mortality dynamics across different months neglects issues regarding seasonality in death counts, which are evident when data are examined by demographers in their countries of interest. Once the dust settled and more researchers were given access to the mortality records, consensus was reached: deaths far exceeded the levels that the government had initially acknowledged (Santos-Lozada & Howard, 2019). At the same time, the spread of misinformation continues to erect walls between scholars and non-academic audiences (Scheufele et al., 2021). Despite efforts to build and reinforce these walls, demographic topics continue to attract the interest of numerous sectors of the population. These new dimensions of demographic research have important implications: every year we observe waves of resistance against sound statistical methods and informed discussions of demographic processes from those who would prefer important and concerning findings to remain in the dark. This challenge is compounded by varying levels of data literacy, including among university graduates from various fields of study.

This shift from the classical academic model—the so-called academic bubble—into a more engaged, participatory, and interactive way of conducting research has challenged demographers to venture into research areas and modes of conduct that had not been previously explored by the founding fathers of the discipline. Young researchers face new responsibilities to define what the “demography of the future” will be, what role demographers should play in the elaboration and discussion of the topics we identify as crucial, and how we can make sure our research penetrates the walls that have been erected between academia, governments, local communities, and the policy world. Only by adopting such reciprocal and participatory approach can our research reach its full potential, ultimately benefiting the individuals who inhabit this world. Similarly, only by changing the way we produce and convey our research can our analyses help subsequent generations better cope with a mutating demographic landscape pervaded by massive threats of misinformation.

The best antidote to misinformation is a clear message on the facts. The COVID-19 pandemic has revealed the importance of demography and demographers in guiding public discussions and improving the usefulness and quality of administrative data. It is no surprise that one of the first articles published on the COVID-19 pandemic was a revisit of basic demographic data and concepts (Dowd et al., 2020). In this article, aptly titled “Demographic science aids in understanding the spread and fatality rates of COVID-19,” the authors convey the clear message that there is a need to go back to the roots of demography to understand how differences in age structures across populations may explain differences in fatality rates across countries. In sharing this “simple” message, the authors called for governments around the world to rapidly mobilize resources and make swift policy decisions to mitigate the pandemic. More recently, demographers highlighted that vaccination rate data are often wrong because many government agencies are often using outdated denominators (Wrigley-Field, 2022; Wrigley-Field et al., 2021). Other valuable strategies to counteract the spread of misinformation come from well-managed and well-informed online forums such as the “Dear Pandemic” initiative, a platform managed by an interdisciplinary team of female researchers and clinicians with varied expertise aimed at educating and empowering individuals to successfully navigate the COVID-19 information overwhelm by providing credible, factual, and timely information.

Engaging in “public demography” does present challenges. The academic system is built on rewarding publications and other academic endeavors. To take this essential step, we must revisit a question asked in the early 2010s: How do we reward public demography (Donaldson, 2011)? Besides community engagement, participatory research, and online dissemination, professional associations may play a stronger role in making demography more visible, accountable, and understandable to the public eye. There is nowadays a very broad range of professional associations that are well positioned to help establish closer connections between scholars and practitioners all over the world, such as Population Association of America (PAA), International Union for the Scientific Study of Population (IUSSP), European Association for Population Studies (EAPS), Canadian Population Society (CPS), Population Europe, Union for African Population Studies (UAPS), and Asian Population Association (APA).

## 6 Summary of Conclusions and Broader Recommendations

In this paper, we have argued that demography is a thriving field and is evolving in multiple promising directions. The uniqueness of demography comes from its analysis of population-level dynamics, its global and international focus, its aim of producing reliable descriptive research and careful population estimates—increasingly coupled with, but not dependent on, experimental and quasi-experimental perspectives—and its focus on, and openness to, innovations in data and measurement. We have also reflected on several directions that we see as promising for demography. From a theoretical and methodological standpoint, given the increasingly “unpredictable” and swift nature of sociodemographic phenomena (e.g., migration and population displacements), a shift in paradigm is needed to keep demography a vibrant, exciting, and policy-relevant field. This shift in paradigm that views demography as a “fast” discipline in turn requires revising demographic theories and methods to keep up with the pace of societal change. From a data standpoint, we have discussed the increasing potential of administrative and longitudinal data sources—especially as the latter become more widely available in LMICs—as well as the power of data interlinkages that allow for granular spatial analyses at the sub-national level. We have also highlighted the promises of big data and computational techniques that increasingly allow scholars to study dynamics in real time (“nowcast”), as well as the rising need for qualitative research and mixed methods. From a pedagogical standpoint, we have stressed the importance of revising the pedagogy of demographic teaching by showcasing the broad applicability of demography methods to topics other than health, mortality, and fertility, and incorporating regular software/coding sessions. Lastly, from a policy perspective, we have discussed the need to make demography more visible and understandable to the public eye, the importance of engaging and co-creating with local communities, and the urge to counteract the spread of misinformation.

On top of these recommendations, we conclude this essay by reflecting on a few additional concrete ways in which our discipline might have a stronger impact both within and outside of academic circles. First, we need faster publication methods and outlets to transform demography into a fast-paced discipline. For instance, the flagship journal of PAA *Demography* could fast track some timely pieces (many journals did this during the COVID-19 pandemic, especially in the fields of epidemiology and public health). Another option is to promote the publication of so-called research notes—i.e., shorter papers highlighting simple yet powerful (and policy-relevant) research findings. There have been improvements on such front; while there were no research notes in the 2019–2021 issues of *Demography*, the 2022 issues included roughly ten research notes, and many other key journals in the field such as *Population Research and Policy Review*, *Demographic Research*, *European Journal of Population*, *Canadian Studies in Population*, and *Population Studies* currently accept research notes.

Second, demographic journals should support a wider range of submissions, i.e., not just manuscripts. In addition to publishing descriptive and causal research articles, research notes, reflections, and reviews, journals should also publish research materials and replication packages, in an effort to boost transparency and replicability within and outside of the discipline, as well as support effective pedagogic practices. As demographers increasingly work with unstructured data, envisioning a repository for demography-related packages and platforms to share code would constitute a huge step forward. For instance, if demographers could easily access demography-related R, Python, or Stata packages and store coding examples in one location, it would be easier for teachers to incorporate novel research frontiers within their demographic method classes and labs. For these innovations to have the strongest possible impact, demographers should strive to build on open science principles that underpin journals’ decisions to offer free Open Access (OA) formats, such as *Demographic Research* and, more recently, *Demography*.

To promote efforts to increase the public face of our field, we should also encourage demographers to write Op-Eds and blog pieces so that our work can reach broader audiences. One such example is the Population Reference Bureau’s US Policy Communications Program, which trains demographers in the third and fourth years of their doctoral programs to effectively communicate research to non-technical audience and policy makers. Other effective outlets are made available by Population Europe and the IUSSP online news magazine, *n-IUSSP*.

Finally, alongside the many panels that IUSSP offers, we recommend the formation of a new IUSSP panel on the pedagogy of the discipline. It has been years since IUSSP created new teaching modules. Developing a shared understanding of what constitutes the core of demographic training would contribute to more cohesion among demographers and lead to an even more vibrant and thriving research field.

## Footnotes

1 May 2022 marked the tenth anniversary of the Centre on Population Dynamics (CPD) at McGill University. On such occasion, a group of scholars and practitioners gathered to celebrate the achievements of the center and reflect upon the current state and future directions of the discipline of demography. As part of the event, four early-career researchers engaged in a panel discussion about the state of the discipline, sharing their personal reflections, hopes, and expectations for the future. This manuscript is a result of such panel and, as such, it summarizes the views and opinions of the panelists.

2 This, in turn, requires publication strategies that may swiftly disseminate key research findings, as discussed in the “Conclusion” section.

3
https://population-europe.eu/events/event-reviews-recordings/third-exploratory-meeting-between-vice-president-european

4
https://www.bls.gov/nls/nlsy26.htm

5 The Canadian Longitudinal Study on Aging (CLSA) is a notable exception.

6
https://www.npr.org/2022/06/24/1102305878/supreme-court-abortion-roe-v-wade-decision-overturn

7
https://www.amnesty.org/en/latest/news/2022/01/poland-regression-on-abortion-access-harms-women/

8
https://edition.cnn.com/2021/04/26/politics/census-us-government-control/index.html

9
https://www.bloomberg.com/news/articles/2015-02-26/the-tragedy-of-canada-s-terrible-census-data
